# Identifying Human Phenotype Terms by Combining Machine Learning and Validation Rules

**DOI:** 10.1155/2017/8565739

**Published:** 2017-11-09

**Authors:** Manuel Lobo, Andre Lamurias, Francisco M. Couto

**Affiliations:** LaSIGE, Faculdade de Ciências, Universidade de Lisboa, Lisboa, Portugal

## Abstract

Named-Entity Recognition is commonly used to identify biological entities such as proteins, genes, and chemical compounds found in scientific articles. The Human Phenotype Ontology (HPO) is an ontology that provides a standardized vocabulary for phenotypic abnormalities found in human diseases. This article presents the Identifying Human Phenotypes (IHP) system, tuned to recognize HPO entities in unstructured text. IHP uses Stanford CoreNLP for text processing and applies Conditional Random Fields trained with a rich feature set, which includes linguistic, orthographic, morphologic, lexical, and context features created for the machine learning-based classifier. However, the main novelty of IHP is its validation step based on a set of carefully crafted manual rules, such as the negative connotation analysis, that combined with a dictionary can filter incorrectly identified entities, find missed entities, and combine adjacent entities. The performance of IHP was evaluated using the recently published HPO Gold Standardized Corpora (GSC), where the system Bio-LarK CR obtained the best *F*-measure of 0.56. IHP achieved an *F*-measure of 0.65 on the GSC. Due to inconsistencies found in the GSC, an extended version of the GSC was created, adding 881 entities and modifying 4 entities. IHP achieved an *F*-measure of 0.863 on the new GSC.

## 1. Introduction

Text mining techniques are essential to deal with the large amount of biomedical literature published every day [1]. One of their contributions is the ability to identify terms in literature that are represented in biomedical ontologies [2]. The Human Phenotype Ontology (HPO) [3] is an ontology that provides a standardized vocabulary for phenotypic abnormalities found in human diseases. The information on this ontology can facilitate the understanding of medical texts, such as electronic health records. However, the recognition of HPO entities in text is a nontrivial task. HPO entities span from simple to highly complex and descriptive entities, which range from 1 to 14 words. Groza et al. [4] provided a study on the complex nature of HPO entities and released a Gold Standard Corpora (GSC) to measure the performance of state-of-the-art Named-Entity Recognition (NER) systems. The top system was Bio-LarK CR that achieved an *F*-measure of 0.56.

This work presents our NER system, dubbed Identifying Human Phenotypes (IHP), which combines machine learning and validation rules to increase the performance to a more acceptable level. IHP adopted the framework provided by IBEnt [5] that uses Stanford CoreNLP [6] for text processing and applies Conditional Random Fields (CRFs) for the identification of entities. IHP uses a rich feature set (linguistic, morphological, orthographic, lexical, context, and other features) based on previous works in Biomedical NER, adapted for the identification of HPO entities. It also applies a validation stage based on manual rules, such as negative connotation analysis, which in combination with a dictionary can remove false positives, identify false negatives, and combine adjacent entities. IHP outperformed Bio-LarK CR in the GSC by achieving an *F*-measure of 0.65. However, to fully understand why IHP did not achieve a larger improvement, we manually analyzed the errors produced by IHP and found some problems in the GSC, mainly missing entities. Thus, we created an extended version of the GSC (GSC+) that added 881 entities and modified 4 entities. Using the GSC+, IHP achieved an *F*-measure of 0.86, which corresponds to a substantial increase (0.30) from the previous top *F*-measure, 0.56. The remainder of this article will detail the data and methods used, the results obtained, and their discussion. Both the GSC+ and IHP source code are available at https://github.com/lasigeBioTM/IHP.

## 2. Materials and Methods

### 2.1. Gold Standard Corpora

In 2015, Groza et al. [4] provided a unique corpus for HPO, dubbed Gold Standard Corpora (GSC). It consists of 228 manually annotated abstracts, containing a total of 1933 annotations and covering 460 unique HPO entities. These 228 abstracts were manually selected to cover 44 complex dysmorphology syndromes analyzed in a previous HPO study [7]. The GSC includes a high diversity of nontrivial matches due to the complexity of the lexical structure of phenotype expressions. For example, in the text “no kidney anomalies were found,” the NER system should be able to recognize the term HP:0000077 (abnormality of the kidney). For each document file in the GSC (one line without a title), there is a corresponding annotation file (as many lines as the number of annotated entities). The annotation is given by three columns: the exact matching character offset, the HPO accession number, and the annotation text (e.g., “[27::42] HP_0000110 ∣ renal dysplasia”).

Besides releasing the GSC, the authors performed a comprehensive evaluation of three NER systems: the NCBO Annotator [8], the OBO Annotator [9], and the Bio-LarK CR [4]. Bio-LarK CR, just like IHP, is a recognition system with the objective of identifying HPO entities, while the NCBO Annotator and the OBO Annotator identify biomedical entities from several ontologies. Of these three annotators, Bio-LarK CR is the only one able to find complex HPO references, since it was developed with HPO as the main target. Thus, as expected, Bio-LarK CR was the top performer achieving an *F*-measure of 0.56, with a recall of 0.49 and a precision of 0.65. However, the performance of Bio-LarK CR was not substantially higher than the OBO Annotator that achieved an *F*-measure of 0.54. Given that these values of *F*-measure are still far from being perfect, there is a need for NER systems that could enhance these levels of performance, for example, by employing machine learning techniques.

### 2.2. HPO Benchmark Annotators

In this work, we implemented three HPO annotators in order to test an updated GSC, the GSC+. We used the OBO Annotator, the NCBO Annotator, and the Minimal Named-Entity Recognizer (MER) [10]. The NCBO Annotator is able to annotate text with relevant ontology concepts from the great number of ontologies available in BioPortal (https://bioportal.bioontology.org/), which is largest repository of biomedical ontologies. We used the NCBO API (http://data.bioontology.org/documentation) targeted towards the HPO. The OBO Annotator is a semantic Natural Language Processing tool capable of combining any number of OBO ontologies from the OBO foundry to identify their terms in a given text. A particular HPO-specific version of this tool was used (available at http://www.usc.es/keam/PhenotypeAnnotation/OBOAnnotatorJAR.zip), which specifically targets HPO entities. To match input text against HPO terms this tool uses two types of prebuilt inverted indexes: lexical and contextual. It provides a graphical interface which allows for an easy annotation of abstracts. MER is a very simple NER tool that given a lexicon (text file with terms representing the entities of interest) and an input text, it returns a list of the annotated entities. It provides an API (available at http://labs.fc.ul.pt/mer/), which already includes some particular lexicons, including the HPO.

To test these annotators, all the abstracts in the GSC were used as the input. The results obtained from each tool were formatted into an identical GSC format and compared to obtain the precision, recall, and *F*-measure. We attempted to execute Bio-LarK CR; however, some of the external links it uses internally are not available anymore. Bio-LarK CR source code has not been updated in the last years, and therefore we were unfortunately unable to make it functional. Nonetheless, it is worth remembering that Bio-LarK CR *F*-measure was only 0.02 higher than OBO Annotator and thus the impact of not using Bio-LarK CR is minimal in our work.

### 2.3. Identification of Human Phenotypes

IHP relies on Stanford CoreNLP [6] and on an implementation of Conditional Random Fields (CRFs) provided by CRFSuite [11]. Biomedical NER systems commonly apply CRFs which are a type of probabilistic model capable of labeling a sequence of tokens (sequence of characters with a specific meaning, such as a word, symbol, or number) and producing an output of annotated named entities (word phrases that can be classified in a particular category). CRFs can include a rich feature set that, given a sequence and the corresponding labels, can obtain the conditional probability of a state sequence (a label) given a certain input sequence. In this case, the label represents the words that are part of a named entity. These models need to be trained on a training set. The trained model is able to label sequences of tokens with the most probable labels, according to that model [12].

CRFSuite was applied with a l2sgd algorithm (Stochastic Gradient Descent with L2 regularization term). The standard algorithm settings of this tool were kept, except for an adjustment of the L1 coefficient value due to a slight improvement in performance and the addition of two Boolean settings (“feature.possible_states” and “feature.possible_transitions”) which affect the way features are generated and can improve the labeling accuracy. The algorithm was used with the following settings:

- *L1 Coefficient* - “0.9833”
- *L2 Coefficient* - “1”
- *feature.possible_states* - “1”
- *feature.possible_transitions* - “1”

**Figure 1:**
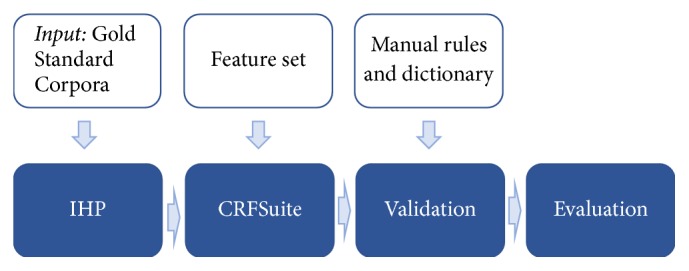
Layout of IHP's annotation pipeline. IHP requires as input a Gold Standard Corpora that will serve as a training set for the CRFSuite and to evaluate IHP performance in the end; a feature set to use in CRFSuite; and a list of rules a dictionary to solve potential errors.

Figure 1 presents the annotation pipeline of IHP. The process starts by loading GSC into IHP to be divided into a training and a testing set. The resulting sets are used to create a model with CRFSuite and a specific feature set. After the annotation process, there is a validation stage, in which a combination of a dictionary and manual rules (e.g., negative connotation analysis) is used to remove false positives, identify missed entities, and combine adjacent entities. In the evaluation stage, the results are calculated, returning the precision and recall of the annotation process.

IHP uses StanfordCoreNLP and GeniaSS [13] (GENIA Sentence Splitter) to preprocess the text. GeniaSS was used with the default parameters. During the training stage, a model was created using CRFSuite, applying a 10-fold cross-validation technique on the GSC. For the creation of the model, a manually crafted feature set (linguistic, orthographic, morphological, context, lexicon, and other features) was selected according to the feature performance and according to the work of previous authors in the area of Biomedical NER [14–21]. This feature set is available at GitHub along with the annotator. The entire feature set includes the following:

- *Linguistic Features*. Lemma and part-of-speech tags of the current token.
- *Orthographic Features*. Word case of the current token and presence of symbols (digits, left bracket, right bracket, slash, dash, quote, double-quote, left parenthesis, right parenthesis) in the token.
- *Morphological Features*. Prefixes (with length from 2 and 3), suffixes (with length from 1 to 4), word shape, and bigrams of the current token.
- *Context Features*. Lemma with a window size of 2, part-of-speech tags with a window size of 4, word shape with a window size of 2, prefixes (with length from 1 and 4) with a window size of 1, and suffixes (with length from 1 and 4) with a window size of 1.
- *Lexical Features*. Stop words with a window size of 4.
- *Other Features*. Brown cluster representation of current token and classification of length class of word.

### 2.4. Validation

The validation stage uses a combination of manual rules and a dictionary to remove false positives, identify missed entities, and combine adjacent entities. The rules and dictionary are available at https://github.com/lasigeBioTM/IHP. The dictionary contains all the terms and synonyms from the HPO database, as well as the training set annotations. The dictionary was processed to increase the amount of entity variations (e.g., for “abnormalities of the kidney” also add “kidney abnormalities” and vice versa). The manual rules work in combination with the dictionary and lists of words. The annotations in the testing set are not used in any of the rules to avoid bias issues. These word lists contain common HPO words, common part-of-speech (POS) tags, and stop words. These word lists can be found at https://github.com/lasigeBioTM/IHP/tree/master/src/other/word_lists.

The rules were developed specifically avoiding the testing set, that is, the rules use information from the training set and the HPO database to create a dictionary, but never from the testing set. The rules can be divided into two categories: identification of entities and removal of entities. The rules of each category will now be described. Further description of how the rules were developed can be found at the GitHub repository pointed above.

#### 2.4.1. Identification of Entities

- *Dictionaries Entities*. It identifies dictionary entities using exact matching.
- *Entity Variations*. It finds specific entity structures by considering a set of common HPO nouns (e.g., “abnormalities” and “malformations”) and possible variations of the next tokens in the sentence (e.g., “of,” “of the,” and “in the”). Using these structures, it then tries to match nouns (e.g., “abnormalities of the ear”) or a group of adjectives (e.g., “defects of the outer, middle, and inner ear”).
- *Longer Entities*. It works similarly to the previous rule; however, instead of finding structures in the sentence, it uses entities from the results set and the dictionary as the base point. After finding these entities in the sentence, it tries to expand the entity boundaries (to the left or to the right) by identifying certain words and POS tags. For example, if “rib anomalies” was previously identified, it would identify “spine and rib anomalies” by expanding to the right, identifying the word “and” and the noun “spine.”
- *Smaller Entities*. It checks if an entity can be separated into more entities by identifying specific words and POS tags (e.g., identification of the entity “pits of the palms” inside “pits of the palms and soles”).
- *Second Validation*. A second validation process is performed using the list of previously identified entities. This list allows the rules to work on a larger number of entities.

#### 2.4.2. Removal of Entities

- *General Errors*. It removes entities with obvious mistakes such as entities formed only by digits, entities containing only a single quote/parenthesis, entities smaller than a total character length of 3, and entities containing more than one specific type of common noun (e.g., “abnormalities” or “malformations”).
- *Incorrect Structure*. It checks the POS tags in an entity and identifies possible errors, removing entities that end in commas, dots, prepositions, and determiners.
- *Negative Connotation Analysis*. It is similar to the Natural Language Processing task of Sentiment Analysis. HPO entities have a negative connotation because they refer to diseases and irregularities. This technique works at a small scale, removing only entities smaller than a length of 3 words. For example, the noun “development” occurs always in conjunction with another word (e.g., “cognitive development”). If this word is found inside an entity, the entity could only have a negative connotation with an additional word (e.g., “cognitive development impairment”). This rule removes entities that follow two conditions: entity has 2 tokens and the entity contains a noun with a positive connotation.
- *Stop Words*. It uses two types of stop word lists with different levels of exclusion. One list contains word phrases that remove entities using exact matching and the other list contains word phrases that remove entities that contain that word phrase in any part of the entity (partial match).

## 3. Results

Using the GSC and a 10-fold cross-validation, IHP achieved an *F*-measure of 0.65.  Table 1 shows that IHP outperforms the comparative annotator in the GSC (increase of 0.09) due to the selected features set and the validation process. It is also important to remember that despite avoiding overfitting IHP was developed using GSC results as a reference.

### 3.1. Feature Performance

To study the feature performance, each feature was tested individually to check its impact. The feature performance results are divided into six categories: linguistic, orthographic, morphological, context, lexical, and others. To minimize the impact of any collinearity issues that may exist we obtained both the results for individual performance in each category, as well as the incremental contribution of each category to the overall performance. The features were ordered from simple features, such as Linguistic and Orthographic, which use the basic structure of a token, to more complex features such as Context and Lexical, which use a larger number of tokens. Although a complete study of feature performance would require every possible combination to be evaluated, we assumed this order to minimize the running time of our experiments. The baseline feature corresponds to the results when only the current token text is considered. Each feature was tested on a single cross-validation iteration. This greedy approach may create some sampling bias and overfitting; however, the objective is to determine which features are more suited for HPO entities and not to analyze the absolute values of precision and recall.

Table 2 shows an improvement in *F*-measure as the features are added to the annotator. This increase in performance is mainly due to an increase in recall since the precision stays relatively the same. Context features have the best performance individually, followed by morphological and linguistic features, and together, they are responsible for most of the performance. Lexical and other features show a smaller effect on the performance.

### 3.2. Validation Rules Performance

During the validation stage, a combination of manual rules and a dictionary was used to correct some of the errors made by the recognition system.  Table 3 shows that although the validation step has a low impact on the *F*-measure, there is a clear increase in the recall and a decrease in precision. This translates to more entities in the GSC being correctly identified but also more word phrases being incorrectly considered as entities. It is possible to see that the identification validation rules are responsible for the increase in recall, while the removal validation rules are responsible for the increase in precision.

### 3.3. GSC+

It is common that some entities in a document may not be detected during the manual annotation process, leaving a gold standard corpus incomplete. This underannotation by the curators may lead to the automatic identification of many false positive which may in fact be correct annotations that were not identified during the manual annotation process, as discussed in [21]. We found that there are some inconsistencies in certain aspects of the GSC, such as the number of times an entity is annotated and the simultaneous annotation of superclass and subclass entities. An example of an inconsistent superclass/subclass entity annotation occurs with the superclass entity “tumours” and the subclass entities “tumours of the nervous system” (document 2888021 in the GSC) and “intracranial tumours” (document 3134615 in the GSC). In the former case, the GSC annotates “tumours” and “tumours of the nervous system” as entities; however, in the latter case, only “intracranial tumours” is annotated.

To understand if there was really an underannotation problem in the GSC, we conducted a test which filters false positives from the results, removing the entities that are not in the GSC test set annotations but that exist in a dictionary containing HPO entities (created from the HPO database and GSC annotations). The filtered false positives are word phrases that should be considered as true positives. Using the previous example, since the entity “tumours” is not considered in the GSC but exists in the HPO dictionary, it would be removed from the results.  Table 4 shows an increase in performance of about 0.17, which is a significant improvement.

To address these issues, we updated GSC, dubbed GSC+ (https://github.com/lasigeBioTM/IHP/blob/master/GSC+.rar) taking into account the inconsistencies found. The GSC+ adds new instances of HPO entities that were automatically identified by IHP. Using the list of identified entities, the entities were checked by exact matching to see if they exist either in the HPO database or in the GSC annotations. The entities that were identified by exact matching were added to the GSC+. The GSC+ provides the addition of 881 new entities and the modification 4 entities. The GSC+ was tested using four different annotators: IHP, OBO Annotator, NCBO Annotator, and MER.  Table 5 shows the results on the GSC+. It shows that IHP has the best performance amongst the annotators, having an *F*-measure 0.31 higher than the best performing annotator.

## 4. Discussion

### 4.1. Feature Performance

Table 2 shows the importance of the selected features and shows that linguistic, morphological, and context features are responsible for the best performance individually and that context features show the best individual performance between the three. The most likely explanation for this is that these features take the neighbor tokens into account and therefore gather more valuable information, allowing the system to perform better using context features over the other types.

The performance improves steadily with the addition of features, focusing mainly on an increase in recall. This increase in recall, but not in precision, means that although more entities are being correctly identified, there are also incorrect word phrases being considered entities.

Before the addition of the features, IHP incorrectly identified word phrases such as “36 schwannomas” (instead of “schwannomas”) and “jerky movements” (instead of “atactic jerky movements”). After the addition of the features, it was able to correct the previous mistakes “schwannomas” and “atactic jerky movements.” Although it corrected some of the errors, it also identified some incorrect word phrases such as “neuroanatomy” and “hippocampus,” probably due to those words being used in other HPO entities.

### 4.2. Validation Performance

The validation process is important for NER systems. We used a combination of manual rules and a dictionary to address some issues of the machine learning classifier. This process removed false positives, identified missed entities, and combined adjacent entities. The developed rules prioritize recall, trying to identify as many entities as possible, according to specific syntactical structures commonly found in HPO entities.* With Identification Rules* leads to an increase of 0.19 in recall in comparison with* No Validation Rules* and remains relatively the same after* With Removal Rules*. Since giving priority to recall leads to the identification of incorrect entities, a removal process is applied afterwards to remove the misidentified entities, improving the precision of the annotator. As seen in the previous example, many of these incorrect word phrases are caused by words that are used in HPO entities such as “hippocampus.” The application of removal rules, such as the use of stop words with different levels of exclusion, helps remove these types of entities. For example, if “hippocampus” is in a stop word list that works with exact matching, it would eliminate the word phrase “hippocampus” but it would not remove a word phrase such as “enlarged hippocampus.” The removal process leads to an increase of 0.08 in precision in comparison with No Validation Rules and an increase of 0.9 in precision after the use of Identification Rules.

Although the *F*-measure remains practically the same before and after the use of all the validation rules, there is a clear increase in recall and decrease in precision. The reason for the low precision values is due to the inconsistent annotation of the GSC and due to IHP's attempt to identify as many HPO entities as possible.

It is important to note that IHP was developed using the GSC results as a reference for the performance, meaning that there is a certain degree of bias. However, the manual rules were developed considering the general HPO entity structure. These rules also try to identify all instances of HPO entities in an abstract, which is something that does not always happen in the GSC. Therefore, we tried to avoid overfitting for this particular dataset when developing the rules, so that IHP can have a similar performance in other contexts.

### 4.3. GSC Inconsistencies

The original GSC contains inconsistencies that could bring confusion to the machine learning-based annotator. By inconsistency we mean similar entity mentions that were annotated differently in multiple locations of a given corpus. The inconsistencies found in the GSC can be divided into four different types: number of annotations, entity meaning, nested entities, and superclass/subclass entities. Some examples of these inconsistencies are presented below.

- *Number of Annotations*. The number of times an entity is identified in a document is inconsistent. For example, the entity “preauricular pits” (document 998578 in the GSC) is used three times during the text and is annotated all three times. In another situation, the entity “medulloblastoma” (document 19533801 in the GSC) is also used three times during the text but it is only annotated twice.
- *Entity Meaning*. In some situations, annotated entities do not exactly match their meaning in the ontology, which can lead to some entities being misidentified. An example is the annotation of “calcium metabolism” (document 6882181 in the GSC) instead of “disturbance of calcium metabolism.” The entity “calcium metabolism” by itself does not have any meaning in the HPO because it does not correspond to any abnormality.
- *Nested Entities*. Nested entities are entities that are contained within other entities. In the GSC some of the entities that are nested inside another entity are annotated while other times they are not. An example of this occurs in the entity “skin and genital anomalies” (document 12219090 in the GSC) and “spine and rib anomalies” (document 9096761 in the GSC). The entity “spine and rib anomalies” is annotated in the GSC, along with the entity “rib anomalies.” However, the same is not true for the entity “skin and genital anomalies.” This entity is annotated in the GSC (accession number: HP_0000078) but the entity “genital anomalies,” which exists in the HPO, is not.
- *Superclass/Subclass Entities*. The final type of inconsistency found has to do with superclass/subclass entities. This is closely related to nested entities because it also involves the identification of entities inside other entities. It is possible that some annotators identify only the most specific class (the subclass) of a certain entity, while others try to identify all the possible classes. However, the GSC is not consistent with the annotation of these types of entities. An example of this occurs with the superclass entity “tumours” and the subclass entities “tumours of the nervous system” (document 2888021 in the GSC) and “intracranial tumours” (document 3134615 in the GSC). In the first case, the GSC annotates both “tumours” and “tumours of the nervous system” as entities. In the second case, only “intracranial tumours” is considered an entity.

### 4.4. GSC+

IHP tries to identify all instances of HPO entities (normal, nested, and subclass/superclass entities), independently of the number of times they appear in the text. Having the correct number of times entities appear in a document can be useful for calculating important values such as the term frequency, which is used to determine the importance of a term in a document. Since IHP tries to annotate as many entities as possible, it will identify a lot of entities that are not in the GSC. This, of course, will cause a decrease in precision and therefore in the overall performance.  Table 4 presents the results from the conducted test to evaluate the potential of IHP in case these inconsistencies were not an issue. Since the test removes from the results all instances of false positives that exist either in the GSC annotations or in the HPO database (by exact matching), there is an increase in precision. The results show that IHP has the potential of achieving an *F*-measure of about 0.82, corresponding to an increase of 0.18 in comparison to the achieved results. This increase suggests that almost a fifth of the annotator's performance can be affected by inconsistencies.

In order to determine IHP's performance in a situation where these inconsistencies were not an issue, the GSC+ was developed in order to provide a more consistent annotation of the abstracts. The development of the GSC+ involves the addition of automatic annotations from the IHP that were matched (with exact matching) with word phrases in the HPO database and GSC annotations. The GSC+ disentangles some of GSC's faults since it adds entities that already were considered HPO entities. Further testing may be conducted in the future to test the effects of the IHP's feature sets and validation step on the GSC+.

The results of IHP in Table 5 were obtained using the same annotation process as with the original GSC. It achieved an *F*-measure of 0.863, an increase of more than 0.04 to its potential performance discussed above, due to the fact that GSC+ includes more improvements than just filtering false positives. IHP had a higher performance than the other annotators on the GSC+. The results show that all three annotators have a lower recall than precision, meaning that they identify a low number of entities in comparison to the total entities in the GSC+. While these annotators all use the HPO as the target ontology to annotate the text, they most likely use exact matching to identify entities in the text and therefore are not prepared for the level of syntactical variation that occurs in HPO entities. Although, IHP is not necessarily capable of identifying entities as long as 14 words, it tries to do it by using validation rules that expand the boundaries of identified entities. Annotators that use exact matching are not able to identify these types of entities since all the words in the entity would have to exactly match the string on the HPO Ontology, which is unlikely. We did not evaluate each validation rule individually as was presented before with GSC, but we expect their impact to be highly similar in GSC+ since we are using the same corpus and ontology.

Another issue of these annotators is the choice of identifying subclass entities over superclass ones. Some annotators, like the OBO Annotator, prefer more specific annotations than more general ones and, therefore, will only identify a portion of those entities.

The reason IHP had a better performance than the other annotators on the GSC+ is that it tries to annotate all instances of HPO entities. We can also see this by comparison of the results in the GSC and the GSC+. There is an increase in precision because all the entities that were previously seen as false positives (and that exist in the HPO database) are now considered true positives.

## 5. Conclusion

We presented IHP, an efficient system for identifying HPO entities in unstructured text, which uses a machine learning-based approach for the identification of entities coupled with a validation technique that combines dictionary-based and manual rules-based methods. IHP outperforms state-of-the-art HPO annotators like Bio-LarK CR in the GSC. This work provides a rich feature set (linguistic, morphologic, orthographic, context, lexical, and other features) for the identification of biomedical entities created based on the work of previous authors and a group of validation rules that are used to fix errors caused by the machine learning-based annotator.

This work also provides an analysis of the inconsistencies found in the GSC. With this analysis, an extended version of GSC, the GSC+, was created which will be left as a contribution. The new annotations were added automatically by using IHP, so GSC+ may still contain some issues, but since they have been exactly matched with curated annotations we believe that there is no much room for errors. This new corpus can be used for further evaluations of HPO annotators and other applications, such as term frequency analysis.

The GSC+ was used to test IHP and other three annotators to provide a more reliable benchmarking tool for HPO annotators. IHP outperformed all three annotators with a substantial performance margin of more than 0.3 in *F*-measure.

In the future, it would be interesting to improve IHP's performance by defining a richer feature set that could allow the identification of more complex entities and by further evaluating and enhancing each validation rule when applied to other domains. It is worth pointing out again that GSC+ is not free of potential misannotations caused by IHP errors, and therefore as future work we aim at conducting a more thorough extension and validation of GSC+ by having the direct contributions from HPO annotators. Furthermore, a larger annotated corpus would allow IHP to deal with a wider number of real-world scenarios. It would also be interesting to apply phenotypic similarity to identify potential misannotations that are not semantically related to the entities found in the text [21]. Since electronic health records contain HPO terms, an exciting challenge would be to assess the performance of IHP in a multilingual corpus [22] and how it could help us to represent their knowledge using linked data technologies [23].

## Figures and Tables

**Table 1:** Comparative performance of IHP and Bio-LarK CR in the Gold Standard Corpora.

	Precision	Recall	*F*-measure
IHP	0.56	0.79	0.65
Bio-LarK CR	0.65	0.49	0.56

**Table 2:** The performance of the different types of used features for IHP: linguistic (L), orthographic (O), morphological (M), context (C), lexical (LE), and others (X). These features were tested in a single cross-validation iteration.

	Precision	Recall	*F*-measure
Baseline	0.452	0.594	0.514
L	0.463	0.72	0.564
O	0.452	0.594	0.514
M	0.457	0.766	0.573
C	0.469	0.783	0.587
Le	0.453	0.606	0.518
X	0.428	0.697	0.530
L + O	0.458	0.720	0.560
L + O + M	0.451	0.760	0.566
L + O + M + C	0.478	0.800	0.598
L + O + M + C + Le	0.478	0.805	0.600
L + O + M + C + Le + X	0.482	0.823	0.608

**Table 3:** Performance of IHP on the Gold Standard Corpora with no validation rules, only identification rules, only removal rules, and all validation rules.

	Precision	Recall	*F*-measure
No Validation Rules	0.672	0.614	0.642
With Identification Rules	0.442	0.797	0.568
With Removal Rules	0.754	0.609	0.674
With Validation Rules	0.549	0.791	0.649

**Table 4:** Potential performance in the Gold Standard Corpora by removing false positives found in either the HPO database or GSC.

	Precision	Recall	*F*-Measure
No Filter	0.549	0.791	0.649
With Filter	0.845	0.791	0.817

**Table 5:** Performance of IHP, OBO Annotator, NCBO Annotator, and MER on the GSC+.

	Precision	Recall	*F*-measure
IHP	0.872	0.854	0.863
OBO Annotator	0.769	0.344	0.475
NCBO Annotator	0.688	0.455	0.548
MER	0.649	0.405	0.499
